# *Lycium barbarum* glycopetide prolong lifespan and alleviate Parkinson’s disease in *Caenorhabditis elegans*

**DOI:** 10.3389/fnagi.2023.1156265

**Published:** 2023-07-04

**Authors:** Jingming Zheng, Zhenhuan Luo, Kin Chiu, Yimin Li, Jing Yang, Qinghua Zhou, Kwok-Fai So, Qin-Li Wan

**Affiliations:** ^1^Department of Pathogen Biology, School of Medicine, Jinan University, Guangzhou, Guangdong, China; ^2^State Key Lab of Brain and Cognitive Sciences, Department of Psychology, The University of Hong Kong, Hong Kong, Hong Kong SAR, China; ^3^Faculty of Medical Science, The Biomedical Translational Research Institute, Jinan University, Guangzhou, Guangdong, China; ^4^Guangdong-Hongkong-Macau Institute of Central Nervous System (CNS) Regeneration, Ministry of Education Central Nervous System (CNS) Regeneration Collaborative Joint Laboratory, Jinan University, Guangzhou, Guangdong, China

**Keywords:** *Lycium barbarum* glycopeptide, *Caenorhabditis elegans*, antiaging, Parkinson’s disease, health span

## Abstract

**Introduction:**

*Lycium barbarum* glycopeptide (LbGp) is the main bioactive compound extracted from the traditional Chinese medicine. *L. barbarum* berries and has been proven to have numerous health benefits, including antioxidative, anti-inflammatory, anticancer, and cytoprotective activities. However, the antiaging effect of LbGp remains unknown.

**Methods:**

The lifespan and body movement of *C. elegans* were used to evaluate the effect of LbGp on lifespan and health span. The thrashing assay was used to determine the role of LbGp in Parkinson’s disease. To investigate the mechanisms of LbGp-induced antiaging effects, we analyzed changes in lifespan, movement, and the expression of longevity-related genes in a series of worm mutants after LbGp treatment.

**Results:**

We found that LbGp treatment prolonged the lifespan and health span of *C. elegans*. Mechanistically, we found that LbGp could activate the transcription factors DAF-16/FOXO, SKN-1/Nrf2, and HSF-1, as well as the nuclear receptor DAF-12, thereby upregulating longevity-related genes to achieve lifespan extension. In addition, we found that the lifespan extension induced by LbGp partially depends on mitochondrial function. Intriguingly, LbGp also ameliorated neurodegenerative diseases such as Parkinson’s disease in a DAF-16-, SKN-1-, and HSF-1-dependent manner.

**Conclusion:**

Our work suggests that LbGp might be a viable candidate for the treatment and prevention of aging and age-related diseases.

## 1. Introduction

Aging, an intrinsic biological process of life, is commonly characterized as a progressive loss of physiological integrity, eventually leading to organ failure (Moskalev et al., 2022). This deterioration of aging is the principal risk factor for many chronic diseases, such as cancer, neurodegenerative disorders, diabetes and cardiovascular diseases (Niccoli and Partridge, 2012). Currently, the percentage of the population over the age of 65 years old has been increasing steadily, which has led to a global burden of age-related chronic diseases (Stegemann et al., 2010). Therefore, slowing the rate of biological aging and the progression of aging-associated diseases will considerably improve the quality of human life. For years, people have been searching for and discovering many natural substances that can prevent aging and aging-related diseases. For example, previous studies have shown that numerous plant extracts and natural bioactive products, such as usnic acid (Xiao et al., 2022), urolithin (Ryu et al., 2016), resveratrol (Park et al., 2012), saponins isolated from *Radix polygalae* (Zeng et al., 2021), and flavonoids from *Lycium barbarum* (*L. barbarum*) leaves (Niu et al., 2022), have lifespan-extending properties in different organism models.

Plant polysaccharides play increasingly important roles in human health and nutrition due to their multiple physiological activities and pharmacological functions (Wang et al., 2020). The berries of *L. barbarum* (*Goji*) (a Solanaceous defoliated shrubbery), a well-known traditional Chinese medicine and super functional food, contain a variety of health-promoting bioactive compounds with numerous nutritional and pharmacological functions (Jin et al., 2013). *L. barbarum* polysaccharides (LBPs) are the main functional constituents of *L. barbarum* and are composed of a variety of acidic heteropolysaccharides and polypeptides or proteins (Masci et al., 2018). Numerous studies have shown different biological activities of LBPs, including antioxidation (Liang et al., 2021), anticancer effects (Mao et al., 2011), antiaging effects (Tang et al., 2019; Zhang et al., 2019), immunoregulation (Ding et al., 2019), reproductive protection, and cytoprotective activity (Zhang et al., 2020). Among them, increasing attention has been given to the functions of LBPs in antioxidative stress and antiaging. Recently, researchers have deeply excavated the main antiaging active ingredients in LBPs and elucidated the underlying molecular mechanisms. For example, Huang W. et al. (2022) showed that the arabinogalactan-protein complex LBGP70 from crude LBPs were able to delay cellular senescence by activating aging-related genes. Zhang et al. (2022) revealed that the acidic heteropolysaccharide LFP-05S from LBPs could prolong the lifespan and enhance stress resistance in *Caenorhabditis elegans* (*C. elegans*) by eliminating unfavorable ROS overproduction.

Recently, researchers further separated and purified LBPs and obtained another component, *L. barbarum* glycopeptide (LbGp), which is the most promising monomeric substance (Peng et al., 2001). LbGp is a kind of glycoprotein whose monosaccharide composition includes glucose, arabinose and galactose and contains 30% protein linked to glycans by O-linkages (Tian, 1995). Accumulating evidence has shown that LbGp protects the kidney, promotes reproduction, enhances immunity, and exerts antitumor and anti-inflammatory effects (Gong et al., 2020; Zhou et al., 2022). The pharmacological functions of LbGp have many similarities with LBPs but also have many differences. For example, previous studies have shown that both LBPs and LbGp can maintain the balance of the intestinal environment by promoting the growth of probiotics, but their effects on the abundance of different types of gut microflora, such as *Akkermansia* and *Alistipes*, are distinctly different (Huang Y. et al., 2022). Therefore, it is valuable to deeply explore the pharmacological activities and underlying molecular mechanisms of LbGp. Considering that LBPs have antioxidant, antiaging and neuroprotective effects, and LbGp is the main active ingredient in LBP, we questioned whether and how LbGp plays a role in ameliorating aging and aging-related disease. Therefore, in this study, we used *C. elegans* to explore the longevity effect of LbGp and its underlying molecular mechanisms.

The nematode *C. elegans* is a robust multicellular model organism with multiple advantages, including a short life cycle, simple physiological structure and easy procedures for manipulation (Wan et al., 2021). Most aging-associated signaling pathways are evolutionarily conserved between *C. elegans* and mammals, including the insulin/insulin-like growth factor signaling (IIS) pathway, dietary restriction (DR)-related pathway, reproductive signaling pathway and mitochondrial dysfunction-related pathways (Xiao et al., 2022). Therefore, *C. elegans* has been widely used in aging research and screening of natural bioactive extracts with antiaging effects (Park et al., 2012; Ryu et al., 2016; Zeng et al., 2021; Xiao et al., 2022).

Our results showed that LbGp significantly increased the lifespan and health span and ameliorated PD-related features in *C. elegans*. Further studies revealed that several aging-related signaling pathways, including the IIS pathway, reproductive signaling pathway and mitochondrial function, were involved in the LbGp-induced longevity effect.

## 2. Materials and methods

### 2.1. Reagents, *C. elegans* strains, and maintenance

*Lycium barbarum* glycopeptide (LbGp) was provided by Ningxia Tianren Goji Biotechnology. LbGp was dissolved in water, and all NGM plates with LbGp were equilibrated overnight before use.

The *Caenorhabditis* Genetics Center (University of Minnesota, Minneapolis, MN, USA) provided the *C. elegans* strains used in this study: wild-type Bristol N2, GR1310 *akt-1(mg144)V*, CB1370 *daf-2(e1370)III*, CF1038 *daf-16(mu86)I*, PS3551 *hsf-1(sy441)I*, CF1903 *glp-1(e2144)III*, AA86 *daf-12(rh61rh411)X*, CB4876 *clk-1(e2519)III*, MQ887 *isp-1(qm150)IV*, RB754 *aak-2(ok524)X*, CF1553 muIs84 [(pAD76)*sod-3p*::GFP + *rol-6(su1006)*], CL2070 dvIs70 [*hsp-16.2p*::GFP + *rol-6(su1006)*], and UM0010 *dat-1p*::GFP; *aex-3p*::α*-syn*(A53T). New strains were generated by standard genetic crosses, and genotypes were confirmed by PCR or sequencing. Here, UM0010 was crossed with CF1038, PS3551, and AA86 to create the double mutants *daf-16(mu86)I; dat-1p*::GFP; *aex-3p*::α*-syn*(A53T), *hsf-1(sy441)I; dat-1p*::GFP; *aex-3p*::α*-syn*(A53T), and *daf-12(rh61rh411)X; dat-1p*::GFP; *aex-3p*::α*-syn*(A53T). All strains were grown and maintained using standard protocols (Jia et al., 2022).

### 2.2. Lifespan assays

All lifespan experiments, except for those with the CF1903 strains, were performed according to the standard protocols at 20°C, as previously described (Jia et al., 2022). In brief, approximately 100–150 young adult worms were picked into fresh plates containing different concentrations of LbGp and 10 μM *5-fluoro-2′-deoxyuridine* (FUDR, Sigma), which was used to prevent progeny production. For CF1903, the synchronized L1 worms were grown at 20°C for 12 h, transferred to 25°C until the young adult stage to eliminate the germline, and finally returned to 20°C for the lifespan assays. For all lifespan assays, heat-inactivated (65°C, 30 min) *Escherichia coli* OP50 was used as food to prevent the metabolism of LbGp by bacteria. Worms were transferred to fresh plates with LbGp every other day. Death events were scored daily, and the lifespan assays were repeated three times. Statistical analyses were performed using the SPSS package, and the *P*-value was determined by the log-rank (Mantel-Cox) method. *P*-value < 0.05 was considered statistically significant. The mean, SEM, *P*-value and lifespan value are summarized in Supplementary Table 1.

### 2.3. Movement assays

The body movement assays were performed using the standard protocol as previously described (Jia et al., 2022). In brief, approximately 150 young adult worms were transferred to plates with or without LbGp at 20°C and maintained as described in the lifespan assays. Then, the movement of the worms was scored daily. When the plates were tapped, the worms moving in a continuous and coordinated sinusoidal pattern were defined to have fast movement; otherwise, they were defined as having non-fast movement.

### 2.4. Thrashing assays

The synchronized L1 PD model worms were grown at 20°C on NGM plates to the young adult stage, transferred to FUDR-containing plates with or without LbGp at 25°C and maintained as described in the lifespan assays. Day 5 worms were picked into a drop of M9 buffer, and the thrashing was recorded for 30 s at × 0.75 magnification with a Motic stereomicroscope as previously described (Huang et al., 2021). At least 30 worms were counted per experiment, and the thrashing assays were repeated three times.

### 2.5. RNA extraction and quantitative RT-PCR

The synchronized L1 worms were grown on plates with or without LbGp to the young adult stage, collected in M9 buffer and washed several times. Worm samples were resuspended using AG RNA^ex^ PRO reagent (Accurate Biology, Changsha, China), and total RNA was isolated by chloroform extraction and isopropanol precipitation. Then, 500 ng RNA was used to synthesize cDNA with a high-capacity cDNA transcription kit (RK20400, ABclonal, Wuhan, China). Quantitative RT-PCR was performed using SYBR Green Select Master Mix (RK21203, ABclonal, Wuhan, China) on a LightCycler480 real-time system (Roche, USA), and each assay was repeated three times. The mRNA expression of the genes was quantified after normalization to the reference gene *cdc-42*, and the *P*-value was computed using the two-tailed Student’s *t*-test. The primers used in this study are shown in Supplementary Table 2.

### 2.6. Fluorescence microscopy and image analyses

For analysis of the fluorescence intensity of *sod-3p*::GFP and *hsp-16.2p*::GFP, the CF1553 and CL2070 strains were grown on plates with or without LbGp to the young adult stage. For CF1553, animals were picked onto 2% agar pads after anesthetizing with 10 μM levamisole, and then, the fluorescence was observed under a Nikon Ti2-U microscope with a 20 × air objective. For CL2070, young adult worms were heated at 35°C for 2 h to stimulate the expression of *hsp-16.2p*::GFP. After recovery at 20°C for 12 h, the CL2070 worms were observed using a Nikon Ti2-U fluorescence microscope with a 20 × air objective. The fluorescence intensity was quantified using ImageJ software. At least 30 animals were used in each group. The *P*-value was calculated by the two-tailed Student’s *t*-test.

### 2.7. Measurement of reactive oxygen species (ROS)

2′7′-Dichlorofluorescein diacetate (H2DCF-DA) was used to detect the levels of intracellular ROS. The synchronized L1 worms were grown on plates with or without LbGp to the young adult stage, transferred to plates with 10 μM H2DCF-DA and incubated for 1 h. In addition, to create oxidative stress conditions, worms were treated with 5 mM paraquat for 12 h before staining with H2DCF-DA. Then, animals were picked onto 2% agar pads after anesthetizing with 10 μM levamisole, and the fluorescence was observed under a Nikon Ti2-U microscope with a 20 × air objective. The fluorescence intensity was quantified using ImageJ software. At least 30 animals were used in each group. The *P*-value was calculated by the two-tailed Student’s *t*-test.

### 2.8. Western blot analyses

CF1553 and CL2070 worms were treated and maintained as described in the section on quantification of fluorescence intensity. Worms were collected in M9 buffer, subjected to three rounds of freezing and thawing, and then lysed in RIPA buffer. The protein samples were boiled at 95°C for 5 min after quantification using a BCA Protein Assay Kit. Next, the protein samples were separated by SDS-PAGE and transferred to PVDF membranes. The membranes were then blocked in 5% milk and incubated with primary antibodies against β-actin (1:5,000, Sigma-Aldrich, A1978) or GFP (1:5,000, Roche, 11814460001). The primary antibodies were visualized by horseradish peroxidase-conjugated anti-mouse secondary antibody and ECL Western Blotting Substrate.

### 2.9. Statistical analyses

The lifespan statistical analyses were performed using the SPSS package, and survival analyses were conducted using the Kaplan-Meier method. *P*-value was determined by log-rank (Mantel-Cox) test for individual experiments. Other data were analyzed by the two-tailed Student’s *t*-test. Statistical significance was defined as *P* < 0.05 and represented as stars (*P < 0.05, ^**^P < 0.01, and ^***^P < 0.001). All experiments were repeated three times independently.

## 3. Results

### 3.1. LbGp enhances lifespan and ameliorates PD-related features in *C. elegans*

**FIGURE 1:**
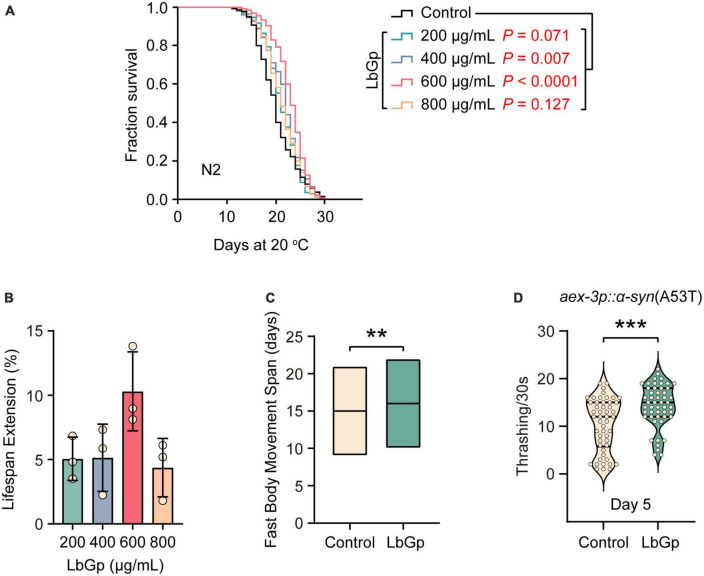
LbGp extended the lifespan and health span of *C. elegans* and improved the body motility of the nematode model of PD. **(A)** Lifespan analyses of N2 worms cultured at different doses of LbGp (200, 400, 600, and 800 μg/mL). **(B)** Statistical analyses of the effect of LbGp on N2 worms lifespan. The data are presented as the mean ± SD of three independent lifespan assays. **(C)** Movement analyses of N2 worms treated with LbGp (600 μg/mL) or vehicle (water). **(D)** Body bend analyses of the PD model strain UM0010 [*aex-3p*::α*-syn*(A53T)] treated with LbGp (600 μg/mL) or vehicle (water) at Day 5. In panels **(A,B)**, lifespan analyses were performed using Kaplan-Meier plotter, and the *P*-value was calculated by the log-rank test. In panels **(C,D)**, mean ± SD, *n* ≥ 30 per group. **P < 0.01, ***P < 0.001 (two-tailed Student’s *t*-test).

To determine whether LbGp plays a role in lifespan regulation, we treated adult worms with different doses of LbGp (200, 400, 600, and 800 μg/mL) and recorded their survival. We found that LbGp dose-dependently enhanced the lifespan of wild-type N2 worms (Figures 1A, B), with 600 μg/mL LbGp showing the best lifespan extension effect (Figures 1A, B). Therefore, 600 μg/mL was selected as the optimal concentration in all subsequent experiments. We also investigated the effect of LbGp on the health span by examining the effect of LbGp on body movement, an aging-related parameter (Huang et al., 2004). The results showed that the period of fast movement was significantly prolonged when worms were treated with LbGp compared with that of the untreated controls (Figure 1C). Taken together, these results demonstrated that LbGp not only prolonged lifespan but also had pronounced health span effects in *C. elegans*, with a concentration of 600 μg/mL showing the best effect.

Given that LbGp can extend lifespan and health span, we asked whether it could have benefits on aging-related diseases, such as Parkinson’s disease (PD). UM0010 is a well-characterized PD model in *C. elegans* by pan-neuron overexpression of human α-synuclein (A53T), which recapitulates some distinct characteristics of PD, including the loss of dopaminergic neurons and motor deficits (Deng and Yuan, 2014). Using UM0010 transgenic worms, we found that LbGp significantly increased the rate of body bending of UM0010 worms at Day 5 (Figure 1D) through a thrashing assay, suggesting that LbGp could ameliorate PD features in *C. elegans*.

### 3.2. LbGp-induced lifespan extension requires the IIS pathway

**FIGURE 2:**
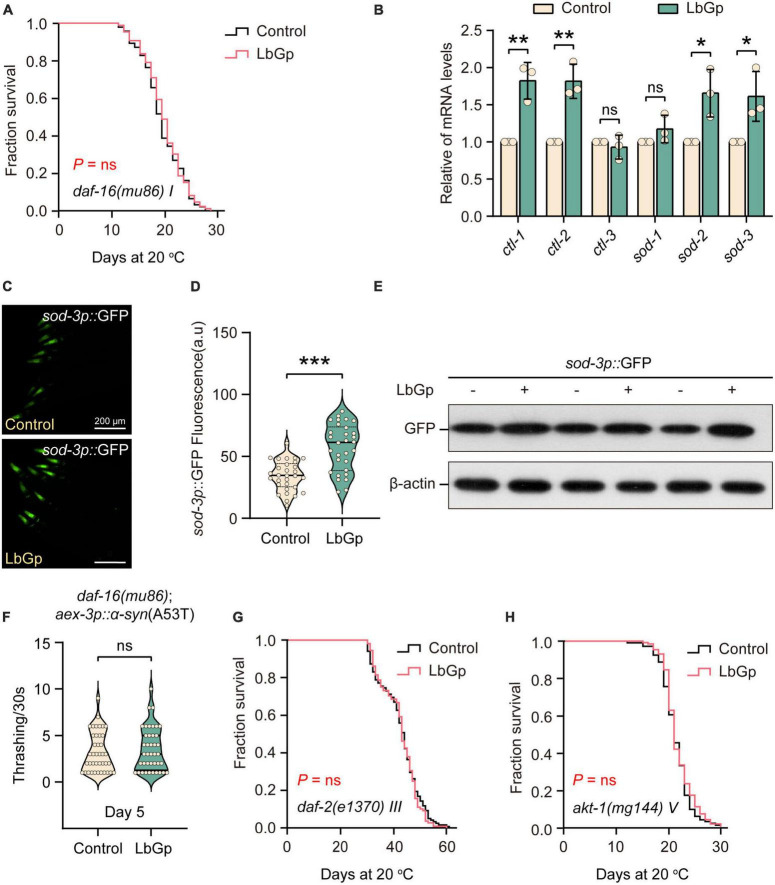
LbGp-induced lifespan extension depended on the IIS pathway. **(A)** Survival curves of *daf-16(mu86)* mutants treated with LbGp (600 μg/mL) or vehicle (water). **(B)** The relative mRNA expression of DAF-16-targeted genes (*ctl-1*, *ctl-2*, *ctl-3*, *sod-1*, *sod-2*, and *sod-3*) in N2 worms treated with LbGp (600 μg/mL) or vehicle (water). **(C,D)** Image **(C)** and quantification **(D)** of GFP fluorescence in the head region of the *sod-3*-reporter strain CF1553 (*sod-3p*::GFP) treated with LbGp (600 μg/mL) or vehicle (water). **(E)** Western blot analyses of GFP from LbGp-treated and non-LbGp-treated CF1553 (*sod-3p*::GFP) transgenic worms. The experiment was repeated three times. The corresponding uncropped western blot figure is shown in Supplementary Figure 1A. **(F)** Body bend analyses of the strain *daf-16(mu86); aex-3p*::α*-syn*(A53T) treated with LbGp (600 μg/mL) or vehicle (water) at Day 5. **(G,H)** Survival curves of *akt-1(mg144)* **(G)** and *daf-2(e1370)* **(H)** mutants treated with LbGp (600 μg/mL) or vehicle (water). In panels **(D,F)**, mean ± SD, *n* ≥ 30 per group. In panels **(B,D,F)**, ns, not significant, *P < 0.05, **P < 0.01, and ***P < 0.001 (two-tailed Student’s *t*-test).

We next determined which molecular mechanisms contribute to the lifespan extension conferred by LbGp. The transcription factor DAF-16, a homolog of human Forkhead box O (FOXO), is one of the main downstream regulators of oxidative stress resistance and the aging process (Li et al., 2019). DAF-16 is the determinant of the longevity effect induced by LBPs and *L. barbarum* berry extracts (Zhang et al., 2019; Xiong et al., 2021). Therefore, we first investigated the role of DAF-16 in the beneficial longevity induced by LbGp. Our results showed that the lifespan of *daf-16(mu86)* worms was not extended by LbGp (Figure 2A). In addition, we found that the transcription levels of the DAF-16 target genes (*ctl-1*, *ctl-2*, *sod-2*, and *sod-3*) were significantly increased by LbGp (Figure 2B). Furthermore, using the GFP fused reporter strain CF1553 (containing a *sod-3p*::GFP fusing transgene), we found that both the fluorescence intensity (Figures 2C, D) and GFP expression level (Figure 2E) were considerably increased when worms treated with LbGP, suggesting that LbGp triggered the expression of *sod-3*, a specific downstream target gene of DAF-16. Consequently, these results indicated that LbGp-induced lifespan extension depends on DAF-16. In addition, we found that LbGp was unable to increase the rate of body bending of UM0010 worms with a *daf-16-null* background (Figure 2F), indicating that similar to lifespan extension, amelioration of PD conferred by LbGp also depends on DAF-16.

Considering that DAF-16 is a key effector downstream of the IIS pathway, we speculated that the LbGp-induced longevity effects might depend on inhibiting the IIS signaling pathway, which is a well-known aging-related signaling pathway (Li et al., 2019). In *C. elegans*, DAF-2 is the insulin/IGF-1 transmembrane receptor homologous to mammals, which cascades to regulate the activity of the phosphoinositide 3-kinase (PI3K)/Akt kinase, eventually leading to the nuclear translocation of DAF-16/FOXO transcription factor and regulating longevity (Li et al., 2019). Using *daf-2(e1370)* and *akt-1(mg144)* mutants, we found that LbGp-induced lifespan extension was abrogated by *daf-2* and *akt-1* mutants (Figures 2G, H), indicating that LbGp-induced lifespan extension was associated with the IIS signaling pathway.

### 3.3. The transcription factors SKN-1/Nrf2 and HSF-1 mediate LbGp-induced longevity

**FIGURE 3:**
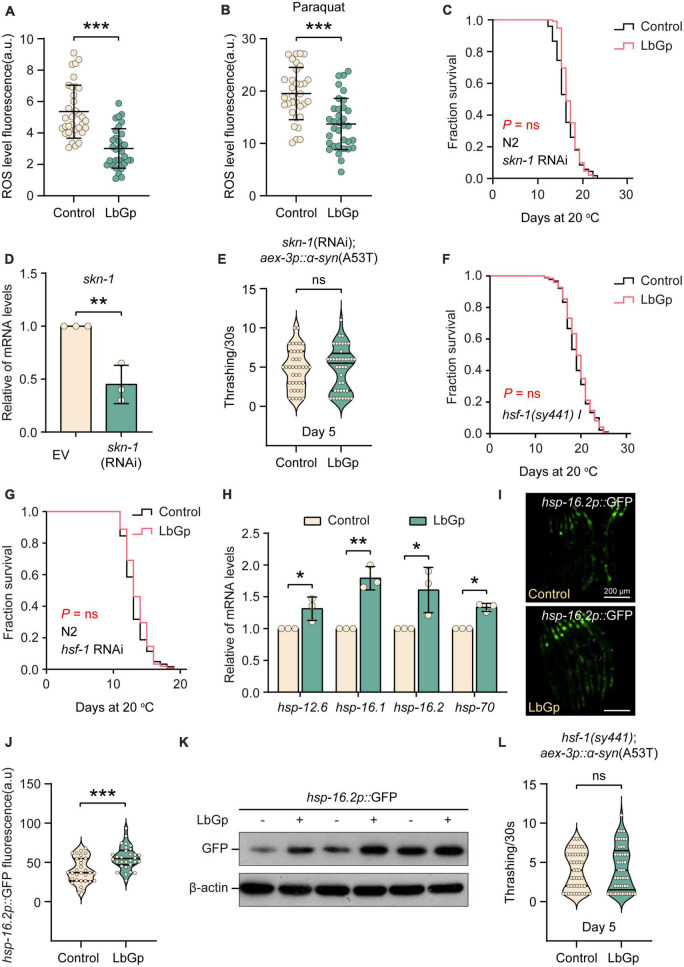
LbGp-induced lifespan extension was mediated by the transcription factors HSF-1 and SKN-1/*Nrf2*. **(A,B)** Quantitation of intracellular levels of ROS in N2 worms treated with LbGp (600 μg/mL) or vehicle (water) under normal conditions **(A)** or oxidative stress conditions (treatment with 5 mM paraquat) **(B)**. **(C)** Survival curves of N2 worms treated with LbGp (600 μg/mL) or vehicle (water) under *skn-1* RNAi conditions. **(D)** Relative *skn-1* mRNA expression in N2 worms fed HT115 bacteria carrying the *skn-1* RNAi vector or empty vector L4440. **(E)** Body bend analyses of the PD model strain UM0010 [*aex-3p*::α*-syn*(A53T)] treated with LbGp (600 μg/mL) or vehicle (water) at Day 5 under *skn-1* RNAi conditions. **(F)** Survival curves of *hsf-1(sy441)* mutants treated with LbGp (600 μg/mL) or vehicle (water). **(G)** Survival curves of N2 worms treated with LbGp (600 μg/mL) or vehicle (water) under *hsf-1* RNAi conditions. **(H)** The relative mRNA expression of *hsf-1*-targeted genes *(hsp-12.6*, *hsp-16.1*, *hsp-16.2*, and *hsp-70*) in the N2 worms treated with LbGp (600 μg/mL) or vehicle (water). **(I,J)** Image **(I)** and quantification **(J)** of GFP fluorescence in the *hsp-16.2*-reporter strain CL2070 (*hsp-16.2p*::GFP) treated with LbGp (600 μg/mL) or vehicle (water). **(K)** Western blot analyses of GFP from LbGp-treated and non-LbGp-treated CL2070 (*hsp-16.2p*::GFP) transgenic worms. The experiment was repeated three times. The corresponding uncropped western blot figure is shown in Supplementary Figure 1B. **(L)** Body bend analyses of the strain *hsf-1(sy441); aex-3p*::α*-syn*(A53T) treated with LbGp (600 μg/mL) or vehicle (water) at Day 5. In panels **(A,B,E,J,L)**, mean ± SD, *n* > 30 per group. In panels **(A,B,D,E,H,J,L)**, ns, not significant, *P < 0.05, **P < 0.01, and ***P < 0.001 (two-tailed Student’s *t*-test).

In addition to DAF-16, the transcription factor SKN-1 is a key longevity regulator downstream of the IIS signaling pathway, which activates various downstream antioxidant and phase II detoxification genes in response to oxidative stress (Tullet et al., 2008). Previous studies have shown that *L. barbarum* extracts and LBPs could activate the antioxidant system of *C. elegans* through the transcription factor SKN-1 to enhance oxidative stress resistance (Meng et al., 2022). We first analyzed the antioxidant activity of LbGp in worms by detecting intracellular ROS accumulation levels using H2DCF-DA (a free radical sensor that can be deacetylated by intracellular esterases to emit fluorescence signals associated with ROS). We found that LbGp treatment significantly reduced the level of ROS under normal conditions (Figure 3A) or oxidative stress conditions (treatment with paraquat) (Figure 3B), suggesting that LbGp has antioxidant capacity to scavenge harmful ROS. Then, we wondered whether SKN-1 functions in lifespan extension induced by LbGp. Conducting survival analyses, we found that similar to the results of *daf-16*, the LbGp-induced survival benefit was blocked by RNAi knockdown of *skn-1* (Figures 3C, D). Moreover, we found that LbGp failed to improve motility in UM0010 worms under *skn-1* RNAi conditions (Figure 3E). Altogether, these results demonstrated that amelioration of aging and aging-related PD conferred by LbGp required the transcription factor SKN-1.

The heat-shock transcription factor HSF-1, another downstream target of the IIS pathway, plays a key role in the lifespan-extending effects of LBPs (Chiang et al., 2012). We also determined the effect of LbGp on the *hsf-1* mutant and found that LbGp-induced lifespan extension was abrogated in the *hsf-1-null* mutant (Figure 3F). Meanwhile, LbGp failed to prolong the lifespan in WT worms under *hsf-1* RNAi conditions (Figure 3G). Subsequently, we also confirmed that the mRNA levels of HSF-1 targets, including *hsp-12.6*, *hsp-16.1*, *hsp-16.2*, and *hsp-70*, were obviously increased by LbGp (Figure 3H). Moreover, we observed an elevated transcription level of *hsp-16.2* by detecting the fluorescence intensity and GFP protein level of the *hsp-16.2p*::GFP transgenic strains in the presence or absence of LbGp (Figures 3I, J, K). Similar to DAF-16 and SKN-1, we also found that the deletion of *hsf-1* eliminated the beneficial effect of LbGp on PD models (Figure 3L). Collectively, these results demonstrated that the LbGp-induced longevity effect was attributed to regulation of the IIS signaling pathway in a DAF-16-, HSF-1- and SKN-1-dependent manner.

### 3.4. LbGp-induced longevity depends on the reproductive signaling pathway

**FIGURE 4:**
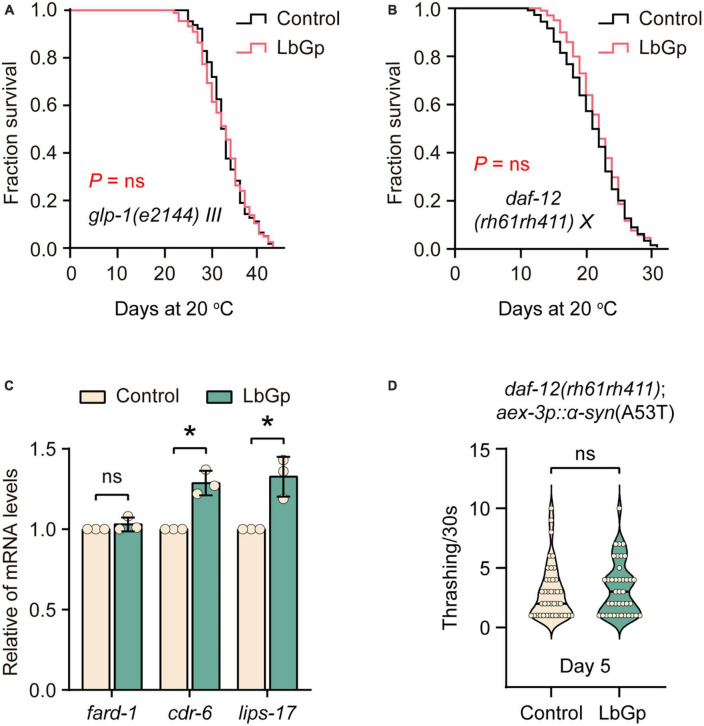
LbGp-induced lifespan extension depended on the reproductive signaling pathway. **(A,B)** Survival curves of *glp-1(e2144)* **(A)** and *daf-12(rh61rh411)* **(B)** mutants treated with LbGp (600 μg/mL) or vehicle (water). **(C)** The relative mRNA expression of the *daf-12*-targeted genes (*fard-1*, *cdr-6* and *lips-17*) in the N2 worms treated with LbGp (600 μg/mL) or vehicle (water). **(D)** Body bend analyses of the strain *daf-12(rh61rh411); aex-3p*::α*-syn*(A53T) treated with LbGp (600 μg/mL) or vehicle (water) at Day 5. In panel **(D)**, mean ± SD, *n* > 30 per group. In panels **(C,D)**, mean ± SD, ns, not significant, *P < 0.05 (two-tailed Student’s *t*-test).

In *C. elegans*, germline depletion extends lifespan by remodeling the transcriptional landscape through activation of several aging-related transcription factors, including DAF-16 and SKN-1 (Berman and Kenyon, 2006). Based on our results above, we questioned whether the LbGp-induced longevity effect was related to the reproductive signaling pathway. Our results showed that LbGp was unable to further increase the lifespan of *glp-1(e2144)* worms, a germline-less and long-lived mutant (Figure 4A). The nuclear steroid receptor DAF-12 is activated by bile acid-like steroids to extend lifespan in germline-less worms (Motola et al., 2006). A recent study showed that DAF-12 contributed to the lifespan extension conferred by LBPs in *C. elegans* (Zhang et al., 2019). Concordantly, we also found that LbGp failed to extend the lifespan of *daf-12(rh61rh411)* (Figure 4B). Additionally, LbGp significantly enhanced the expression of DAF-12 target genes (*cdr-6* and *lips-17*) (Figure 4C). Furthermore, we found that improvement of motility of UM0010 worms was disappeared in a *daf-12-null* background (Figure 4D). Altogether, these findings illustrated that the survival benefits induced by LbGp were mediated by the reproductive signaling pathway.

### 3.5. LbGp-induced longevity partially depends on mitochondrial function

**FIGURE 5:**
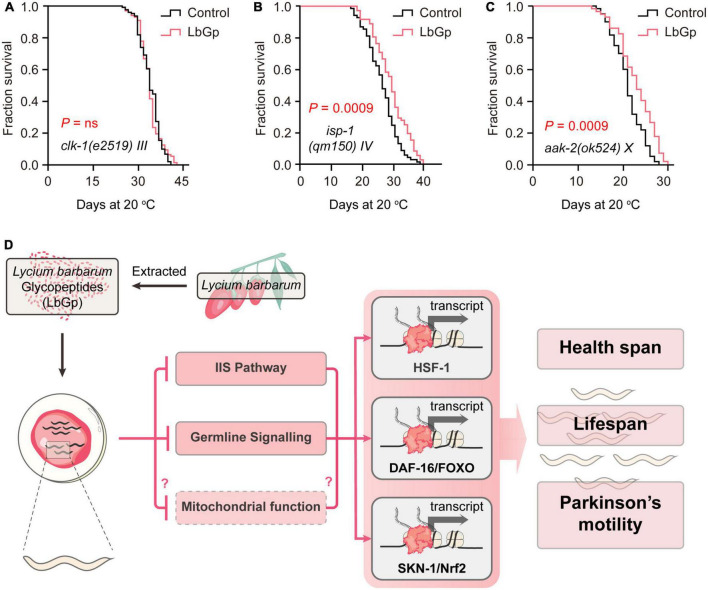
LbGp-induced lifespan extension partially depended on mitochondrial function. **(A–C)** Survival curves of *clk-1(e2519)* **(A)**, *isp-1(qm150)* **(B)** and *aak-2(ok524)* **(C)** mutants treated with LbGp (600 μg/mL) or vehicle (water). **(D)** Mechanisms of action of LbGp in *C. elegans*.

Attenuated mitochondrial respiratory function and mitochondrial dysfunction are major causes of aging. SKN-1 has been shown to improve mitochondrial function by regulating mitochondrial biogenesis, ultimately extending the lifespan of *C. elegans* (Palikaras et al., 2015). Thus, we further investigated the effect of LbGp on the long-lived mitochondrial mutants *clk-1(e2519)* and *isp-1(qm150)*. *clk-1* encodes the human coenzyme Q7 hydroxylase homolog, and *isp-1* is the Rieske iron-sulfur polypeptide 1 homolog in *C. elegans*. Our results showed that the lifespan of *clk-1(e2519)* was unable to be extended by LbGp (Figure 5A), but that of *isp-1(qm150)* could be (Figure 5B), suggesting that the LbGp-induced longevity effect required mitochondrial function in a *clk-1*-specific manner.

Mitochondrial are the central organelle of energy production. The discovery of a mechanism by which LbGp regulates lifespan associated with mitochondrial function motivated us to deduce whether LbGp prolongs lifespan by influencing energy generation. In *C. elegans*, *aak-2* encodes a catalytic subunit of AMP-activated kinase (AMPK), which senses energy levels and is activated by a low ATP level to regulate lifespan (Apfeld et al., 2004). We found that LbGp could further extend the lifespan of the *aak-2* mutant (Figure 5C), suggesting that *aak-2* is dispensable for the survival advantage induced by LbGp. Therefore, this result indicated that LbGp-induced lifespan extension was independent of the regulation of energy production.

## 4. Discussion

Aging is always accompanied by a deterioration of physiological function and an increase in degenerative disease (e.g., Alzheimer’s disease and Parkinson’s disease) (Niccoli and Partridge, 2012). As the global population ages, the need to find substances that can treat aging-related diseases, delay the aging process, and prolong lifespan is urgent. Functional and nutraceutical foods have gradually attracted attention because of their few side effects. *L. barbarum* has been recognized and used as traditional Chinese medicine for 2,500 years, and its use is now expanding to all Western countries, where it is consumed mostly as food supplements (Amagase and Farnsworth, 2011). In this study, we found that LbGp, a potential ingredient of *L. barbarum* berry, could extend lifespan and health span, as well as alleviate the progression of aging-related PD. In an in-depth study of the mechanism, we found that the amelioration of aging and aging-related disease conferred by LbGp was mediated by regulating the IIS signaling pathway and reproductive signaling pathway, subsequently activating the stress response transcription factors DAF-16, HSF-1 and SKN-1 and the nuclear receptor DAF-12, thereby triggering the expression of downstream longevity-related target genes to extend lifespan. Furthermore, mitochondrial function was involved in the survival benefits induced by LbGp (Figure 5D).

The imbalance between the production and elimination of free radicals is one of the major factors in aging and aging-associated disorders, and the supplementation of antioxidants can delay aging and improve oxidative stress resistance (Hekimi et al., 2011). Previous studies have shown that LBPs have antioxidant bioactivity and can reduce DNA damage by eliminating free radicals and inhibiting oxidative stress (Zhang et al., 2017). Consistently, we also found that LbGp could activate the expression of several antioxidant genes. Moreover, we confirmed that SKN-1, a major oxidative stress response factor, contributes to LbGp-induced lifespan extension. These results demonstrated that, similar to LBPs, LbGp prolonged lifespan depending on its antioxidant bioactivity, at least in part. Indeed, we also found that LbGp extended lifespan in an antioxidant-independent manner, that is, regulation of *glp-1* by LbGp. The longevity of germline-deficient mutants is associated with increased ROS in the soma (Wei and Kenyon, 2016). However, we found that LbGp, an antioxidant, did not influence the lifespan of *glp-1(e2144)* worms, which seems to contradict our observation that LbGp improves the antioxidant capacity. These findings implied that LbGp-induced lifespan extension in *C. elegans* depended not only on the antioxidant capacity but also on other biological activities.

Aging is the greatest risk factor for most age-related disorders, and delaying the rate of biological aging through the consumption of antiaging drugs or foods is able to slow the onset and progression of age-related diseases (Xiao et al., 2022). In the present study, we found that LbGp could improve motility in a PD model of *C. elegans* through mechanisms consistent with antiaging, indicating that LbGp can ameliorate the progression of PD. In support of our findings, previous studies reported that the crude extracts and polysaccharides of *L. barbarum* berry have protective effects against many aging-associated disorders, including cardiovascular diseases and neurodegenerative diseases. For example, supplementation with LBPs protected neurons against β-amyloid-induced apoptosis and improved the learning and memory abilities of Sprague Dawley rats with scopolamine-induced brain injury; crude extracts from LB can alleviate the neurotoxicity of α-synuclein protein, β-amyloid, and Aβ peptide *in vitro* and *in vivo* (Meng et al., 2022). Those studies and our present results indicated that LbPs could improve the symptoms of neurodegenerative disorders and that LbGp may be the one of the main active ingredients in LBPs exert these protective effects.

## 5. Conclusion

In conclusion, the innovation of this study lies in the discovery that LbGp extracted from *L. barbarum* berries can prolong the lifespan and health span and delay the occurrence of aging-related diseases such as PD in *C. elegans*. These findings provide a new strategy for preventing aging and aging-related diseases. Mechanistically, the molecular mechanism of LbGp-induced longevity is closely related to the IIS pathway, reproductive signaling pathway and mitochondrial function-related signaling pathway. It is necessary to expand our findings on LbGp to mammalian model organisms. Therefore, in future research, we will continue to search for new protective effects against other neurodegenerative diseases and clarify the molecular mechanisms of LbGp against aging and aging-related diseases in different animal models.

## Data availability statement

All data needed to evaluate the conclusions in this manuscript are present in this manuscript and/or the Supplementary material. Requests to access the datasets should be directed to Q-LW, wanqinli@hotmail.com.

## Author contributions

JZ: investigation, methodology, software, visualization, and writing–editing. ZL: investigation, methodology, visualization, and writing–original draft. KC: investigation, methodology, and visualization. YL and JY: methodology. QZ: investigation and funding acquisition. K-FS: investigation and writing–reviewing and editing. Q-LW: investigation, writing–reviewing and editing, and funding acquisition. All authors contributed to the article and approved the submitted version.
