# Supplementary material for: Lycium barbarum glycopetide prolong lifespan and alleviate Parkinson’s disease in Caenorhabditis elegans
Source: Front Aging Neurosci. 2023 Jul 4;15:1156265. doi: 10.3389/fnagi.2023.1156265 (PMC10353607; doi:10.3389/fnagi.2023.1156265)
Supplement: Supplementary file 1 [file Data_Sheet_1.docx]

Supplementary Material

*Lycium barbarum* Glycopetide Prolong Lifespan and Alleviate Parkinson's disease in *Caenorhabditis elegans*

**Jingming Zheng ^1, †^, Zhenhuan Luo ^1, †^, Kin Chiu ^2, †^, Yimin Li ^1^, Jing Yang ^3^, Qinghua Zhou ^3^, Kwok-Fai So ^4, *^, Qin-Li Wan ^1, *^**

*** Correspondence:** Qin-Li Wan: wanqinli@hotmail.com; Kwok-Fai So: [hrmaskf@hku.hk](mailto:hrmaskf@hku.hk)

# Supplementary Figure 1. Uncropped western blot figure.


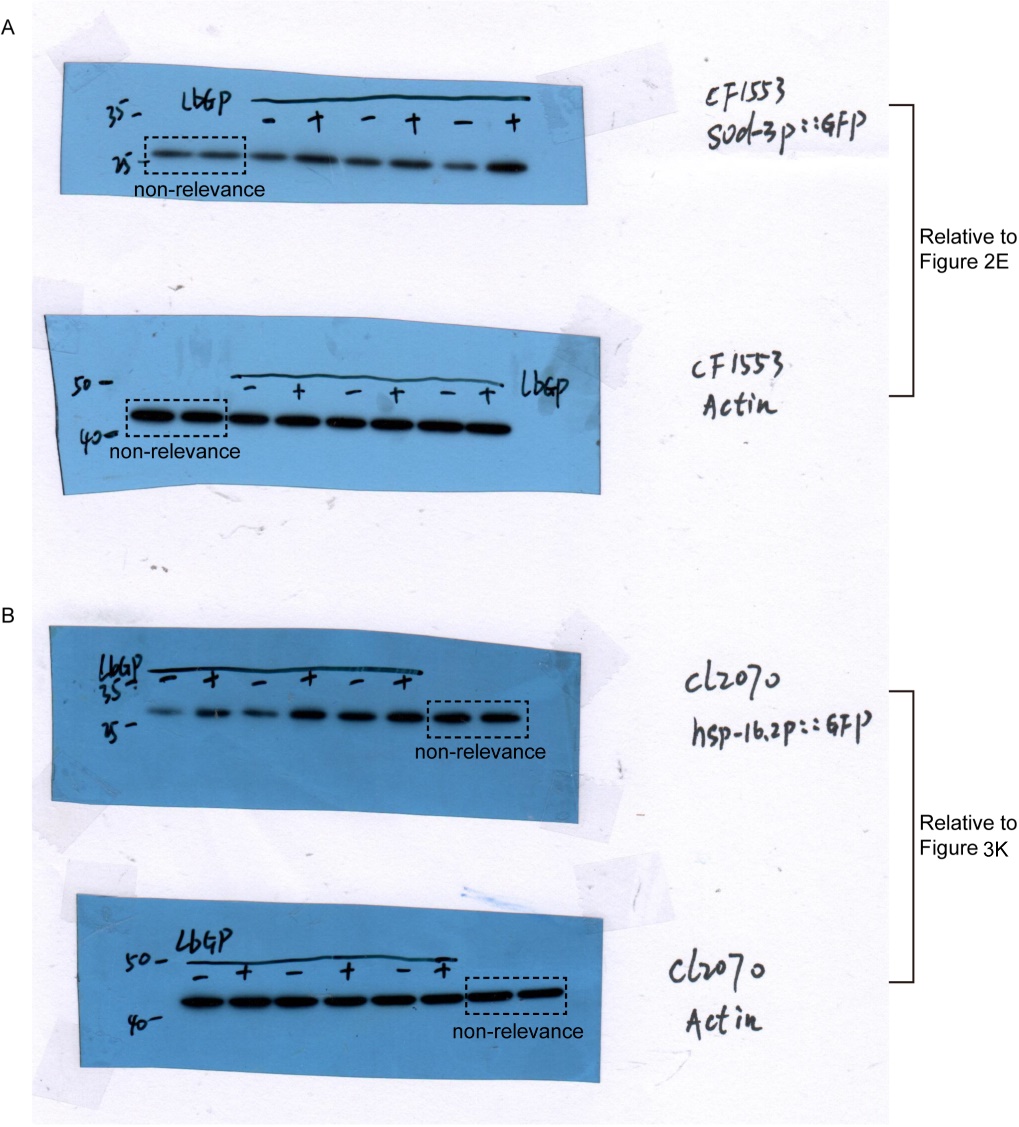


# Supplementary Table 1. The lifespan data and statistics.

| **Figure** | **Strains** | **Treatments** | **Mean**  **Lifespan ± SEM**  **(days)** | | **P-value**  **VS**  **control** | **%**  **Change in mean lifespan** | | **N** |  |
| --- | --- | --- | --- | --- | --- | --- | --- | --- | --- |
| **Figure 1A&1B** | **N2(WT)** |  |  | |  |  | |  |  |
|  | EXP. 1 | 20 ℃/control | 20.164±0.339 | | \ | \ | | 140 |  |
|  |  | 20 ℃/200 μg/mL LbGp | 21.135±0.266 | | 0.197 | 4.815 | | 170 |  |
|  |  | 20 ℃/400 μg/mL LbGp | 21.638±0.323 | | 0.007 | 7.309 | | 152 |  |
|  |  | 20 ℃/600 μg/mL LbGp | 22.948±0.285 | | <0.0001 | 13.806 | | 135 |  |
|  |  | 20 ℃/800 μg/mL LbGp | 21.212±0.297 | | 0.127 | 5.195 | | 151 |  |
|  | EXP. 2 | 20 ℃/control | 21.362±0.335 | | \ | \ | | 127 |  |
|  |  | 20 ℃/200 μg/mL LbGp | 22.110±0.382 | | 0.071 | 3.501 | | 118 |  |
|  |  | 20 ℃/400 μg/mL LbGp | 22.614±0.299 | | 0.007 | 5.858 | | 163 |  |
|  |  | 20 ℃/600 μg/mL LbGp | 23.096±0.339 | | <0.0001 | 8.118 | | 135 |  |
|  |  | 20 ℃/800 μg/mL LbGp | 22.667±0.389 | | 0.007 | 6.106 | | 105 |  |
|  | EXP. 3 | 20 ℃/control | 21.286±0.334 | | \ | \ | | 126 |  |
|  |  | 20 ℃/200 μg/mL LbGp | 22.742±0.271 | | 0.006 | 6.843 | | 163 |  |
|  |  | 20 ℃/400 μg/mL LbGp | 21.761±0.265 | | 0.490 | 2.233 | | 180 |  |
|  |  | 20 ℃/600 μg/mL LbGp | 23.193±0.246 | | <0.0001 | 8.961 | | 202 |  |
|  |  | 20 ℃/800 μg/mL LbGp | 21.667±0.283 | | 0.724 | 1.790 | | 135 |  |
|  |  |  |  | |  |  | |  |  |
| **Figure 2A** | **CF1038 *daf-16(mu86)I*** | |  | |  |  | |  |  |
|  | EXP. 1 | 20 ℃/control | 18.968±0.279 | | \ | \ | | 186 |  |
|  |  | 20 ℃/600 μg/mL LbGp | 19.287±0.281 | | 0.562 | # | | 171 |  |
|  | EXP. 2 | 20 ℃/control | 18.723±0.304 | | \ | \ | | 155 |  |
|  |  | 20 ℃/600 μg/mL LbGp | 18.989±0.255 | | 0.799 | # | | 189 |  |
|  | EXP. 3 | 20 ℃/control | 19.194±0.296 | | \ | \ | | 134 |  |
|  |  | 20 ℃/600 μg/mL LbGp | 19.766±0.301 | | 0.073 | # | | 158 |  |
|  |  |  |  | |  |  | |  |  |
| **Figure 2G** | **CB1370 *daf-2(e1370)III*** | | |  | | |  | |  |
|  | EXP. 1 | 20 ℃/control | 42.515±0.615 | | \ | \ | | 132 |  |
|  |  | 20 ℃/600 μg/mL LbGp | 43.576±0.618 | | 0.339 | # | | 125 |  |
|  | EXP. 2 | 20 ℃/control | 42.483±0.700 | | \ | \ | | 118 |  |
|  |  | 20 ℃/600 μg/mL LbGp | 42.361±0.663 | | 0.573 | # | | 108 |  |
|  | EXP. 3 | 20 ℃/control | 43.886±0.750 | | \ | \ | | 123 |  |
|  |  | 20 ℃/600 μg/mL LbGp | 44.469±0.552 | | 0.425 | # | | 128 |  |
|  | EXP. 4 | 20 ℃/control | 43.841±0.607 | | \ | \ | | 138 |  |
|  |  | 20 ℃/600 μg/mL LbGp | 43.247±0.833 | | 0.777 | # | | 93 |  |
|  |  |  |  | |  |  | |  |  |
| **Figure 2H** | **GR1310 *akt-1(mg144)V*** | | |  | | |  | |  |
|  | EXP. 1 | 20 ℃/control | 21.283±0.273 | | \ | \ | | 106 |  |
|  |  | 20 ℃/600 μg/mL LbGp | 21.736±0.225 | | 0.289 | # | | 129 |  |
|  | EXP. 2 | 20 ℃/control | 21.315±0.295 | | \ | \ | | 111 |  |
|  |  | 20 ℃/600 μg/mL LbGp | 21.240±0.312 | | 0.829 | # | | 104 |  |
|  | EXP. 3 | 20 ℃/control | 22.258±0.504 | | \ | \ | | 62 |  |
|  |  | 20 ℃/600 μg/mL LbGp | 22.254±0.470 | | 0.997 | # | | 71 |  |
|  |  |  |  | |  |  | |  |  |
| **Figure 3C** | **N2(WT) *skn-1* RNAi** | | |  | | |  | |  |
|  | EXP. 1 | 20 ℃/control | 16.068±0.284 | | \ | \ | | 74 |  |
|  |  | 20 ℃/600 μg/mL LbGp | 16.809±0.176 | | 0.156 | # | | 115 |  |
|  | EXP. 2 | 20 ℃/control | 16.096±0.230 | | \ | \ | | 83 |  |
|  |  | 20 ℃/600 μg/mL LbGp | 16.273±0.173 | | 0.697 | # | | 128 |  |
|  | EXP. 3 | 20 ℃/control | 15.724±0.236 | | \ | \ | | 87 |  |
|  |  | 20 ℃/600 μg/mL LbGp | 15.797±0.217 | | 0.837 | # | | 74 |  |
|  |  |  |  | |  |  | |  |  |
| **Figure 3F** | **PS3551 *hsf-1(sy441)I*** | |  | |  |  | |  |  |
|  | EXP. 1 | 20 ℃/control | 19.020±0.334 | | \ | \ | | 99 |  |
|  |  | 20 ℃/600 μg/mL LbGp | 19.299±0.301 | | 0.772 | # | | 107 |  |
|  | EXP. 2 | 20 ℃/control | 18.856±0.269 | | \ | \ | | 111 |  |
|  |  | 20 ℃/600 μg/mL LbGp | 19.323±0.286 | | 0.223 | # | | 99 |  |
|  | EXP. 3 | 20 ℃/control | 19.056±0.300 | | \ | \ | | 90 |  |
|  |  | 20 ℃/600 μg/mL LbGp | 19.415±0.253 | | 0.457 | # | | 123 |  |
|  |  |  |  | |  |  | |  |  |
| **Figure 3G** | **N2(WT) *hsf-1* RNAi** | |  | |  |  | |  |  |
|  | EXP. 1 | 20 ℃/control | 13.521±0.147 | | \ | \ | | 144 |  |
|  |  | 20 ℃/600 μg/mL LbGp | 13.564±0.154 | | 0.743 | # | | 149 |  |
|  | EXP. 2 | 20 ℃/control | 13.269±0.155 | | \ | \ | | 134 |  |
|  |  | 20 ℃/600 μg/mL LbGp | 13.333±0.149 | | 0.844 | # | | 132 |  |
|  | EXP. 3 | 20 ℃/control | 13.146±0.160 | | \ | \ | | 123 |  |
|  |  | 20 ℃/600 μg/mL LbGp | 13.539±0.139 | | 0.103 | # | | 152 |  |
|  | EXP. 4 | 20 ℃/control | 13.092±0.142 | | \ | \ | | 131 |  |
|  |  | 20 ℃/600 μg/mL LbGp | 13.411±0.158 | | 0.126 | # | | 129 |  |
|  | EXP. 5 | 20 ℃/control | 13.322±0.134 | | \ | \ | | 174 |  |
|  |  | 20 ℃/600 μg/mL LbGp | 13.468±0.174 | | 0.555 | # | | 111 |  |
|  | EXP. 6 | 20 ℃/control | 12.807±0.109 | | \ | \ | | 181 |  |
|  |  | 20 ℃/600 μg/mL LbGp | 12.909±0.115 | | 0.502 | # | | 187 |  |
|  |  |  |  | |  |  | |  |  |
| **Figure 4A** | **CF1903 *glp-1(e2144)III*** | | |  | | |  | |  |
|  | EXP. 1 | 20 ℃/control | 31.569±0.626 | | \ | \ | | 58 |  |
|  |  | 20 ℃/600 μg/mL LbGp | 32.789±0.515 | | 0.147 | # | | 95 |  |
|  | EXP. 2 | 20 ℃/control | 32.625±0.529 | | \ | \ | | 88 |  |
|  |  | 20 ℃/600 μg/mL LbGp | 32.938±0.544 | | 0.981 | # | | 64 |  |
|  | EXP. 3 | 20 ℃/control | 32.045±0.753 | | \ | \ | | 66 |  |
|  |  | 20 ℃/600 μg/mL LbGp | 32.190±0.669 | | 0.642 | # | | 58 |  |
|  |  |  |  | |  |  | |  |  |
| **Figure 4B** | **AA86 *daf-12(rh61rh411)X*** | | |  | | |  | |  |
|  | EXP. 1 | 20 ℃/control | 20.265±0.511 | | \ | \ | | 98 |  |
|  |  | 20 ℃/600 μg/mL LbGp | 20.214±0.452 | | 0.582 | # | | 98 |  |
|  | EXP. 2 | 20 ℃/control | 20.412±0.412 | | \ | \ | | 96 |  |
|  |  | 20 ℃/600 μg/mL LbGp | 21.638±0.371 | | 0.104 | # | | 94 |  |
|  | EXP. 3 | 20 ℃/control | 21.150±0.447 | | \ | \ | | 107 |  |
|  |  | 20 ℃/600 μg/mL LbGp | 21.970±0.398 | | 0.495 |  | | 99 |  |
|  |  |  |  | |  |  | |  |  |
| **Figure 5A** | **CB4876 *clk-1(e2519)III*** | | |  | | |  | |  |
|  | EXP. 1 | 20 ℃/control | 33.175±0.309 | | \ | \ | | 126 |  |
|  |  | 20 ℃/600 μg/mL LbGp | 33.081±0.359 | | 0.981 | # | | 99 |  |
|  | EXP. 2 | 20 ℃/control | 33.059±0.651 | | \ | \ | | 68 |  |
|  |  | 20 ℃/600 μg/mL LbGp | 33.677±0.542 | | 0.869 | # | | 65 |  |
|  | EXP. 3 | 20 ℃/control | 31.965±0.606 | | \ | \ | | 86 |  |
|  |  | 20 ℃/600 μg/mL LbGp | 33.368±0.501 | | 0.454 | # | | 76 |  |
|  |  |  |  | |  |  | |  |  |
| **Figure 5B** | **MQ887 *isp-1(qm150)IV*** | | |  | | |  | |  |
|  | EXP. 1 | 20 ℃/control | 26.986±0.602 | | \ | \ | | 69 |  |
|  |  | 20 ℃/600 μg/mL LbGp | 29.847±0.650 | | 0.0009 | 10.9 | | 72 |  |
|  | EXP. 2 | 20 ℃/control | 27.047±0.550 | | \ | \ | | 64 |  |
|  |  | 20 ℃/600 μg/mL LbGp | 29.292±0.520 | | 0.012 | 8.3 | | 65 |  |
|  | EXP. 3 | 20 ℃/control | 26.354±0.528 | | \ | \ | | 82 |  |
|  |  | 20 ℃/600 μg/mL LbGp | 28.097±0.536 | | 0.013 | 6.6 | | 93 |  |
|  |  |  |  | |  |  | |  |  |
| **Figure 5C** | **RB754 *aak-2(ok524)X*** | | |  | | |  | |  |
|  | EXP. 1 | 20 ℃/control | 21.100±0.441 | | \ | \ | | 60 |  |
|  |  | 20 ℃/600 μg/mL LbGp | 22.965±0.561 | | 0.0009 | 10.9 | | 57 |  |
|  | EXP. 3 | 20 ℃/control | 21.342±0.309 | | \ | \ | | 79 |  |
|  |  | 20 ℃/600 μg/mL LbGp | 22.279±0.375 | | 0.023 | 4.4 | | 68 |  |
|  | EXP. 4 | 20 ℃/control | 20.219±0.469 | | \ | \ | | 64 |  |
|  |  | 20 ℃/600 μg/mL LbGp | 21.936±0.427 | | 0.009 | 8.5 | | 78 |  |

**Table S1** The lifespan data and statistics. All statistical analyses were performed using the SPSS package, and survival analyses were conducted using the Kaplan-Meier method. P-value was determined by log-rank (Mantel-Cox) test for individual experiments. “N”: the number of dead worms. “#”: no calculate (because p > 0.05).

# Supplementary Table 2. The primers used in qRT-PCR

| **Gene** |  | **Primer sequences (5’-3’)** |
| --- | --- | --- |
| *cdc-42* | Forward | CTGCTGGACAGGAAGATTACG |
|  | Reverse | CTCGGACATTCTCGAATGAAG |
| *ctl-1* | Forward | GACGTATCCAAAACCCCAAGTG |
|  | Reverse | TTGGCATGAACGACACGCTC |
| *ctl-2* | Forward | TTCCGATCGAGGACTCCCAG |
|  | Reverse | CTTCACTCCTTGAGTTGGCTTG |
| *ctl-3* | Reverse | CCCACATGGTCAATCTAACGGT |
|  | Forward | GGAGCTCCATTGGATGTGGT |
| *sod-1* | Forward | CGTAGGCGATCTAGGAAATGTG |
|  | Reverse | AACAACCATAGATCGGCCAACG |
| *sod-2* | Forward | AGCTTTCGGCATCAACTGTC |
|  | Reverse | AAGTCCAGTTGTTGCCTCAAGT |
| *sod-3* | Forward | AGCATCATGCCACCTACGTGA |
|  | Reverse | CACCACCATTGAATTTCAGCG |
| *hsp-12.6* | Forward | GTGATGGCTGACGAAGGAAC |
|  | Reverse | GGGAGGAAGTTATGGGCTTC |
| *hsp-16.1* | Forward | GTCACTTTACCACTATTTCCGTCCAGCTCAACGTTC |
|  | Reverse | CAACGGGCGCTTGCTGAATTGGAATAGATCTTCC |
| *hsp-16.2* | Forward | CTGCAGAATCTCTCCATCTGAGTC |
|  | Reverse | AGATTCGAAGCAACTGCACC |
| *hsp-70* | Forward | AATGAACCAACTGCTGCTGCTCTT |
|  | Reverse | TGTCCTTTCCGGTCTTCCTTTTG |
| *fard-1* | Forward | GGGTTTTTGGGAAAGGTGAT |
|  | Reverse | CCACCGATTGCTTTCAATTT |
| *cdr-6* | Forward | TCGGGCTTCTCGGTTTACC |
|  | Reverse | CAGCTTTGACCAGAGGAACCA |
| *lips-17* | Forward | ATCTGTTGCTGGAGCCAATCG |
|  | Reverse | TATCCAACTTTATCGTCTCC |
